# Benefits of sacubitril/valsartan use in patients with chronic heart failure after cardiac valve surgery: a single-center retrospective study

**DOI:** 10.1186/s13019-023-02252-y

**Published:** 2023-04-11

**Authors:** Jian Zheng, Qingsong Wu, Qianzhen Li, Mirong Tang, Jian He, Zhihuang Qiu, Linfeng Xie, Liangwan Chen

**Affiliations:** 1 Department of Cardiovascular Surgery, Union Hospital, Fujian Medical University, Xinquan Road 29, 350001 Fuzhou, Fujian P. R. China; 2 Department of Pharmacy, The First Affiliated Hospital of Fujian Medical University, Fuzhou, Fujian P. R. China; 3 Key Laboratory of Cardio-Thoracic SurgeryFujian Medical University), Fujian Province University, Fujian Medical University), Fuzhou, Fujian P. R. China; 4 Fujian Medical University, Fuzhou, Fujian P. R. China

**Keywords:** Cardiac valve surgery, Chronic heart failure, Left ventricular ejection fraction, Sacubitril/valsartan, Benefits

## Abstract

**Objectives:**

To evaluate the efficacy of sacubitril/valsartan for the treatment of patients with chronic heart failure (CHF) after cardiac valve surgery (CVS).

**Methods:**

Data were collected from 259 patients who underwent CVS due to valvular heart disease and were admitted to the hospital with CHF from January 2018 to December 2020. The patients were divided into Group A (treatment with sacubitril/valsartan) and Group B (treatment without sacubitril/valsartan). The duration of treatment and follow-up was 6 months. The two groups’ prior and clinical characteristics, post-treatment data, mortality, and follow-up data were analysed.

**Results:**

The effective rate of Group A was higher than that of Group B (82.56% versus 65.52%, P < 0.05). The left ventricular ejection fraction (LVEF, %) was improved in both groups. The final value minus the initial value was (11.14 ± 10.16 versus 7.15 ± 11.18, P = 0.004). The left ventricular end-diastolic/-systolic diameter (LVEDD/LVESD, mm) in Group A decreased more than in Group B. The final value minus the initial value was (-3.58 ± 9.21 versus − 0.27 ± 14.44, P = 0.026; -4.21 ± 8.15 versus − 1.14 ± 12.12, P = 0.016, respectively). Both groups decreased the N-terminal prohormone of B-type natriuretic peptide (NT-proBNP, pg/ml). The final value minus initial value was [-902.0(-2226.0, -269.5) versus − 535.0(-1738, -7.0), P = 0.029]. The systolic and diastolic blood pressure (SBP/DBP, mmHg) in Group A decreased more than in Group B. The final value minus the initial value was (-13.13 ± 23.98 versus − 1.81 ± 10.89, P < 0.001; -8.28 ± 17.79 versus − 2.37 ± 11.41, P = 0.005, respectively). Liver and renal insufficiency, hyperkalaemia, symptomatic hypotension, angioedema, and acute heart failure had no statistical differences between the two groups.

**Conclusions:**

Sacubitril/valsartan can effectively improve the cardiac function of patients with CHF after CVS by increasing LVEF and reducing LVEDD, LVESD, NT-proBNP, and BP, with good safety.

## Background

Currently, the incidence of heart failure (HF) is increasing worldwide. HF with a reduced ejection fraction (HFrEF) has various causes [1, 2]. It is increasingly considered to be a syndrome with multiple systemic mechanisms, including the inflammation and disorders of the neurohormonal system, renin–angiotensin–aldosterone system (RAAS), natriuretic peptide systems (NPs), endothelium, autonomic nervous system, and vasopressin system [3, 4]. In clinical trials, these drug treatments have been shown to help reduce morbidity and mortality progressively [5]. Severe valvular heart disease is associated with an increased risk of morbidity and mortality but can be successfully treated with surgery. Current guidelines recommend the mitral valve and/or aortic valve repair in symptomatic patients with left ventricular ejection fraction (LVEF) > 30% and asymptomatic patients with left ventricular dysfunction (LVEF ≤ 60% and/or left ventricular end-systolic diameter (LVESD) ≥ 45 mm) [6–10]. Despite adherence to current recommendations and successful surgery, valvular heart disease after cardiac valve surgery (CVS) can still occur with left ventricular dysfunction and progressive progression to chronic heart failure (CHF) over long-term follow-up [11, 12].

Previous studies have revealed that blocking RAAS decreases morbidity and mortality in patients with HFrEF [13–15]. However, the efficacy of this treatment for patients with CHF following CVS is relatively unknown. Therefore, for patients with CHF, as a complication of CVS, further foundational research on multi-neurohormone pathways, multi-system mechanisms, and cytokine activation methods is necessary [16–18]. The ACC/AHA/HFSA Focus Update on New Drug Therapy for Heart Failure provides guidelines for new therapies against CHF, including the use of angiotensin receptor-neprilysin inhibitors (ARNI) (sacubitril/valsartan) [19]. In this study, sacubitril/valsartan was used to treat patients with CHF after CVS. The purpose of this study was to investigate the clinical effect of sacubitril/valsartan in the treatment of CHF after CVS by analyzing the prior-treatment clinical characteristics, post-treatment data, mortality, and follow-up data of patients in the treatment and non-treatment groups.

## Methods

### Patient population and data collection

Data were collected from 259 patients who underwent CVS owing to valvular heart disease and were admitted to the Union Hospital, Fujian Medical University, with CHF, from January 2018 to December 2020. The patients were divided into Group A (172 cases) and Group B (87 cases) based on whether the patients were regularly treated with sacubitril/valsartan.

#### Inclusion criteria

1. CVS owing to valvular heart disease, without treatment with sacubitril/valsartan before surgery.
2. Meeting the relevant diagnostic criteria for CHF according to the ESC Guidelines for the Diagnosis and Treatment of Acute and Chronic Heart Failure 2016 [20].

#### Exclusion criteria

1. Coronary heart disease with an organic valvular disease requiring revascularisation, severe arrhythmia, acute HF, and cardiogenic shock.
2. Contraindication to the drug used in this study.
3. Liver disease requiring plasma exchange and renal failure requiring hemodialysis, accompanied by other serious systemic diseases.
4. The presence of serious organic valvular lesions that require more surgical treatment after initial surgery, artificial valve disorders or valve ageing.

After admission, all patients were treated with angiotensin-converting enzyme inhibitor (ACEI)/angiotensin receptor blockers (ARB), mineralocorticoid receptor antagonists, β-blockers, and diuretics for conventional anti-HF as per their clinical conditions. Conventional anti-HF therapy was defined as the treatment regimen recommended according to the ‘2016 ESC Guidelines for diagnosing and treating acute and chronic heart ‘failure’, including ACEI or ARB [20]. After discharge, the original treatment plan was administered, and patients were regularly followed up for half a year. In addition to conventional treatment, the patients in Group A were treated with sacubitril/valsartan (oral administration of an initial dose of 24/26 mg, twice daily, and gradually increasing to 97/103 mg, depending on the follow-up blood pressure) (Entresto, Approval number: H20170363, Novartis Pharma Schweiz AG, Specifications: 100 mg [sacubitril 49 mg/valsartan 51 mg]). The patients in Group B were treated only with conventional anti-HF therapy. The duration of treatment and follow-up was 6 months. The two groups’ prior and clinical characteristics, post-treatment data, mortality, and follow-up data were analysed (Tables 1, 2, and 3). This study was approved by the ethics committee of Union Hospital, Fujian Medical University, and conformed to the Declaration of Helsinki.

**Table 1:** Preoperative and intraoperative data on the two patient groups

Valuables	Group A(n = 172)	Group B(n = 87)	P value
**Valve diseases** ^*^			
Mitral stenosis(n, %)	53 (30.81)	33 (37.93)	0.251
Mitral insufficiency(n, %)	75(43.60)	38 (43.68)	0.991
Aortic stenosis(n, %)	66(38.37)	40 (45.98)	0.240
Aortic insufficiency(n, %)	68(39.53)	32 (36.78)	0.667
Tricuspid insufficiency(n, %)	58(33.72)	30 (34.48)	0.903
Preoperative EF (%)	60.73 ± 6.37	60.08 ± 8.25	0.523
**Surgical procedure**			
Aortic valve	41 (23.84)	23 (26.44)	0.852
Mitral valve	55 (31.98)	21 (24.14)	0.417
Tricuspid valve	5 (2.91)	2 (2.30)	0.954
Combined valvular surgery	71 (41.27)	41 (47.12)	0.537
**Surgical data**			
Operative time (min)	149.73 ± 34.34	147.01 ± 36.05	0.554
Circulation bypass time(min)	70.55 ± 19.56	72.63 ± 20.09	0.424
Aortic Clamp time (min)	37.59 ± 6.37	37.82 ± 14.28	0.900

Continuous variables were present as mean ± SD or median and inter-quartile range, and the counts were expressed as a percentage.Chi-square test for categorical variables and t test or wilcoxon rank sum test for continuous variables. The following tables show the same expression

*Valve diseases was defined as moderate or severe valve stenosis or regurgitation

**Table 2:** Prior treatment data on the two patient groups

Valuables	Group A(n = 172)	Group B(n = 87)	P value
Age(years)	65.1 ± 7.5	66.0 ± 7.1	0.355
Male gender(n, %)	73 (42.44)	49 (56.32)	0.095
Body mass index(kg/m^2^)	23.52 ± 2.72	23.88 ± 2.52	0.314
Time interval the first surgery(years)	11.0(6.0, 14.0)	12.0(7.0, 13.0)	0.903
Heart rate(beats/minute)	83.3 ± 13.9	84.4 ± 17.8	0.623
Cardiac function grade			
NYHA I(n, %)	0 (0.00)	0 (0.00)	N/A
NYHA II(n, %)	25(14.53)	11(12.64)	0.918
NYHA III(n, %)	133 (77.33)	68 (78.16)	0.995
NYHA IV(n, %)	14 (8.14)	8 (9.20)	0.985
Hypertension(n, %)	68 (39.53)	40 (45.98)	0.496
SBP(mmHg)	127.28 ± 19.75	125.11 ± 15.60	0.470
DBP(mmHg)	78.80 ± 15.97	79.28 ± 14.60	0.127
Atrial fibrillation(n, %)	34 (19.77)	11 (12.64)	0.521
Diabetes mellitus(n, %)	36 (20.93)	14 (16.09)	0.680
Coronary heart disease(n, %)	11 (6.40)	6 (6.80)	0.972
LVEF(%)	37.92 ± 4.68	36.90 ± 5.91	0.162
LEVDD(mm)	54.13 ± 6.90	54.87 ± 6.80	0.415
LVESD(mm)	42.67 ± 7.82	43.86 ± 7.45	0.242
Left atrial size(mm)	44.00 ± 7.74	42.69 ± 7.10	0.186
Cardiac output(L/min)	5.0(3.5, 6.0)	5.0(4.1, 5.5)	0.415
Artificial valve disorder(n, %)	0 (0.00)	0 (0.00)	N/A
Mild mitral insufficiency(n, %)	5 (2.91)	2 (2.30)	0.904
Moderate mitral insufficiency(n, %)	0 (0.00)	0 (0.00)	N/A
Severe mitral insufficiency(n, %)	0 (0.00)	0 (0.00)	N/A
Mild tricuspid insufficiency(n, %)	7 (4.07)	3 (3.45)	0.923
moderate tricuspid insufficiency(n, %)	2 (1.16)	1 (1.15)	0.545
Severe tricuspid insufficiency(n, %)	0 (0.00)	0 (0.00)	N/A
Hemoglobin(g/L)	122.93 ± 17.25	122.84 ± 14.90	0.967
Hematocrit(%)	39.46 ± 6.33	38.87 ± 4.08	0.396
NT-ProBNP(pg/ml)	1399.0(775.0, 2814.0)	1028.0(769.0, 2307.0)	0.249
serum creatinine(umol/L)	75.80 ± 28.32	78.70 ± 32.39	0.459
Alanine aminotransferase(IU/L)	28.31 ± 24.73	30.29 ± 27.87	0.382
Aspartate aminotransferase(IU/L)	33.55 ± 23.57	33.70 ± 23.96	0.960
Blood glucose(mmol/L)	5.47 ± 1.66	5.36 ± 1.43	0.573
Potassium ion(mmol/L)	4.05 ± 0.50	3.95 ± 0.50	0.157
Sodium ion(mmol/L)	140.51 ± 3.33	141.03 ± 2.29	0.140
Length of stay(d)	9.9 ± 5.1	11.1 ± 5.8	0.075

Continuous variables were present as mean ± SD or median and inter-quartile range, and the counts were expressed as a percentage. Chi-square test for categorical variables and t test or wilcoxon rank sum test for continuous variables. The following tables show the same expression

**Table 3:** Follow-up data on the two patient groups

Valuables	Group A(n = 172)	Group B(n = 87)	P value
Effective therapy(n, %)	142 (82.56)	57 (65.52)	**<**0.001
**Cardiac function grade**			
NYHA I (n, %)	48 (27.91)	18 (20.69)	0.727
NYHA II (n, %)	85 (49.42)	36 (41.38)	0.359
NYHA III (n, %)	33 (19.19)	26 (29.89)	0.274
NYHA IV (n, %)	6 (3.49)	7 (8.05)	0.724
Heart rate(beats/minute)	81.49 ± 13.65	83.49 ± 11.50	0.241
SBP(mmHg)	114.15 ± 13.01	123.31 ± 12.54	<0.001
SDP(mmHg)	70.52 ± 8.53	76.91 ± 12.28	<0.001
LVEF(%)	49.06 ± 9.96	44.05 ± 9.67	0.005
LEVDD(mm)	50.55 ± 9.13	54.60 ± 12.67	0.004
LVESD(mm)	38.46 ± 6.61	42.72 ± 9.04	<0.001
Left atrial size(mm)	44.00 ± 8.54	43.31 ± 8.16	0.533
Cardiac output(L/min)	5.3(4.2, 6.5)	5.3(4.8, 6.4)	0.180
Hemoglobin(g/L)	121.08 ± 16.34	119.39 ± 16.13	0.430
Hematocrit(%)	38.65 ± 5.26	38.22 ± 4.08	0.473
NT-ProBNP(pg/ml)	612.0(399.0, 788.0)	668.0(435.0, 891.5)	0.014
Serum creatinine(umol/L)	75.67 ± 17.14	76.23 ± 17.69	0.807
Alanine aminotransferase(IU/L)	30.40 ± 9.31	30.98 ± 9.89	0.649
Aspartate aminotransferase(IU/L)	31.56 ± 9.38	30.82 ± 9.70	0.550
Blood glucose(mmol/L)	5.21 ± 1.52	5.55 ± 1.61	0.095
Potassium ion(mmol/L)	4.20 ± 0.38	4.21 ± 0.34	0.807
Sodiumion(mmol/L)	139.80 ± 4.55	139.35 ± 4.44	0.452
Liver insufficiency(n, %)	0 (0.00)	0 (0.00)	N/A
Renal insufficiency(n, %)	0 (0.00)	0 (0.00)	N/A
Hyperkalemia(n, %)	0 (0.00)	0 (0.00)	N/A
Symptomatic hypotension(n, %)	0 (0.00)	(0.00)	N/A
Angioedema(n, %)	0 (0.00)	0 (0.00)	N/A
Acute heart failure(n, %)	3 (1.74)	2 (2.30)	0.864
Follow-up death(n, %)	0 (0.00)	0 (0.00)	N/A

Continuous variables were present as mean ± SD or median and inter-quartile range, and the counts were expressed as a percentage.Chi-square test for categorical variables and t test or wilcoxon rank sum test for continuous variables. The following tables show the same expression

#### Outcome measures

A comparative evaluation was conducted on the clinical treatment effect of the two groups according to NYHA classification. Effectiveness was defined as an NYHA cardiac function grade improved by 1 grade or more. An invalid outcome was defined as no significant change or decrease in NYHA cardiac function.

Effective rate = Effectiveness/total quantity*(100%).

NYHA cardiac function grade, systolic blood pressure (SBP), diastolic blood pressure (DBP), resting heart rate, hypertension, diabetes mellitus, coronary heart disease, and atrial fibrillation were recorded prior to and post-treatment in both groups. The time interval between the first surgery and morning fasting venous blood samples were taken to measure and compare the N-terminal prohormone of B-type natriuretic peptide (NT-proBNP), serum creatinine, alanine aminotransferase, aspartate aminotransferase, potassium ion, sodium ion, haemoglobin, haematocrit, and blood glucose levels. Echocardiography was performed in the two groups, and a comparative analysis was performed on the changes in cardiac function indicators in the two groups, including LVEF, LVEDD, LVESD, left atrial size, and cardiac output. During the treatment, adverse drug reactions, hospitalisation time, readmission times due to cardiac insufficiency, and acute HF cases were observed in the two groups.

#### Statistical analysis

All statistical analyses were performed using SPSS version 19.0 (SPSS, Inc., Chicago, IL, USA). Data types included measurement, enumeration, and grade data. Distributed variables were presented as mean ± standard deviation (SD) or median and inter-quartile range. Categorical variables were presented as numbers and percentages (n, %). The comparisons of measurements were performed with a t-test or the Mann–Whitney test, where appropriate. P-values < 0.05 were statistically significant.

## Results

The NYHA cardiac function classification was between grade II and IV, including 36 cases of grade II, 201 cases of grade III, 22 cases of grade IV, and LVEF between 25% and 50% (Table 2).

Group A included 73 men and 114 women, with an average age of 65.1 ± 7.5 years. The time interval of the first surgery was 11.0(6.0,14.0) years. Group B included 49 men and 38 women, with an average age of 65.1 ± 7.5 years, and the time interval of the first surgery was 11.0(6.0,14.0) years. There was no significant difference in baseline data between the two groups (P > 0.05), which were comparable (Table 2).

**Fig. 1:**
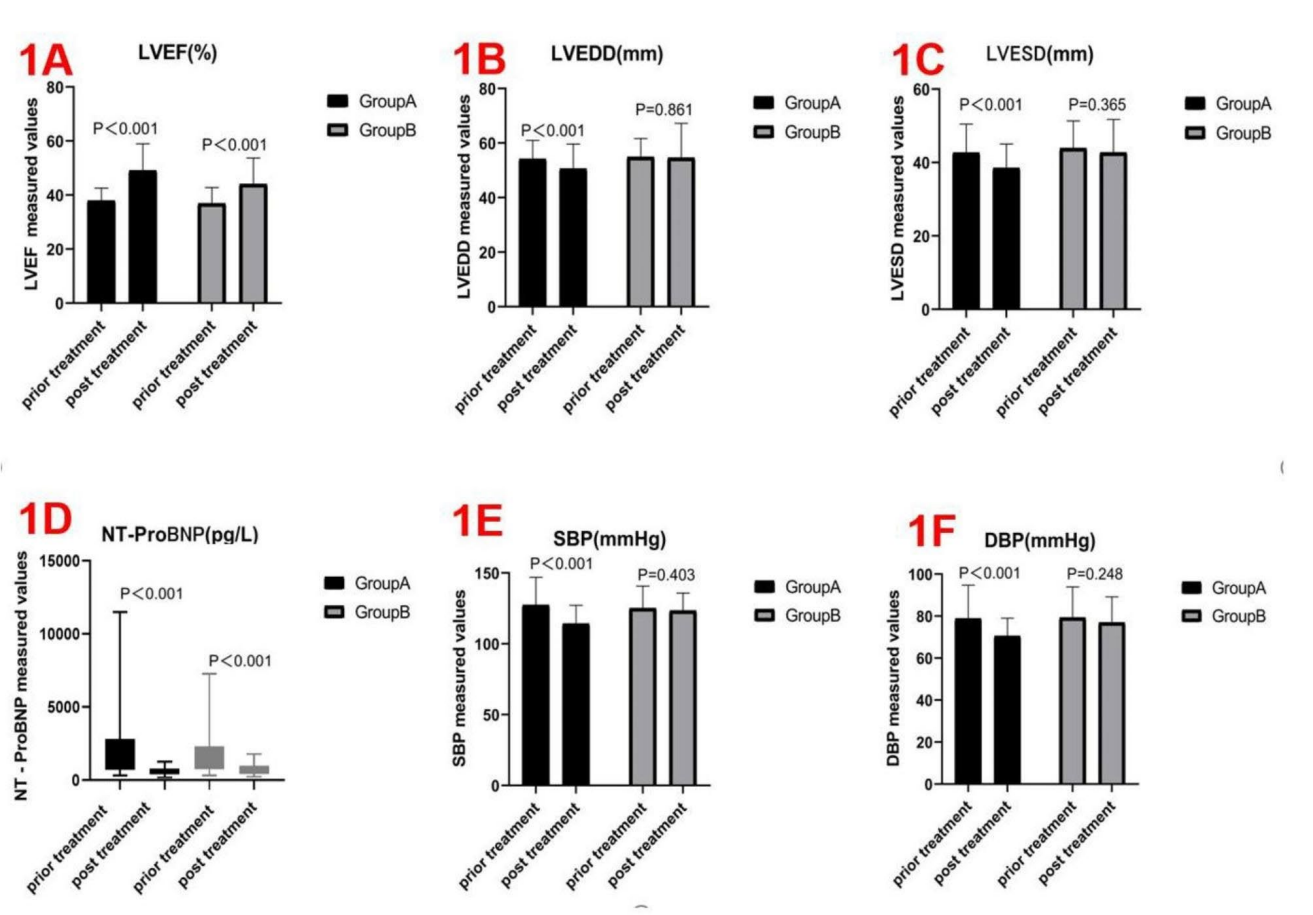
Comparison data of intra-group differences prior and post treatment on the two patient groups

1. Clinical effect: The total effective rate of Group A was higher than that of Group B (82.56% versus 65.52%, P < 0.001) (Table 3).
2. The LVEF improved in both groups; however, Group A exhibited significantly more improvement than Group B in the final value minus the initial value (11.14 ± 10.16% versus 7.15 ± 11.18%, P = 0.004). The LVEDD/LVESD in Group A decreased more than that in Group B in final value minus initial value (-3.58 ± 9.21 mm versus − 0.27 ± 14.44 mm, P = 0.026; -4.21 ± 8.15 mm versus − 1.14 ± 12.12 mm, P = 0.016, respectively). There was no difference in left atrial size and cardiac output between the two groups before and after treatment (P > 0.05; Tables 3 and 4; Fig. 1A and B C).

3. The NT-proBNP in both groups decreased; however, the decrease was greater in Group A than in Group B in final value minus initial value [-902.0(-2226.0, -269.5) pg/ml versus − 535.0(-1738, -7.0) pg/ml, P = 0.029]. There was no significant difference in serum creatinine, alanine aminotransferase, aspartate aminotransferase, potassium ion, sodium ion, haemoglobin, haematocrit, and blood glucose levels between the two groups (P > 0.05). (Tables 3 and 4; Fig. 1D )
4. The SBP and DBP in Group A decreased more than those in Group B in final value minus initial value (-13.13 ± 23.98 mmHg versus − 1.81 ± 10.89 mmHg, P < 0.001; -8.28 ± 17.79 mmHg versus − 2.37 ± 11.41 mmHg, P = 0.005, respectively). (Tables 3 and 4; Fig. 1E F)
5. During follow-up, no adverse reactions, such as liver and renal insufficiency, hyperkalaemia, symptomatic hypotension, and angioedema, were observed in both groups. No deaths occurred. Three patients in Group A and two in Group B were hospitalised for acute HF(Table 3).

**Table 4:** Comparison data of inter-group differences prior and post treatment on the two patient groups

Valuables	Group A(n = 172)	Group B(n = 87)	P value
∆ SBP(mmHg)	-13.13 ± 23.98	-1.81 ± 10.89	<0.001
∆ DBP(mmHg)	-8.28 ± 17.79	-2.37 ± 11.41	0.005
∆ LVEF(%)	11.14 ± 10.16	7.15 ± 11.18	0.004
∆ LVEDD(mm)	-3.58 ± 9.21	-0.27 ± 14.44	0.026
∆ LVESD(mm)	-4.21 ± 8.15	-1.14 ± 12.12	0.016
∆ NT-ProBNP(pg/ml)	-902.0(-2226.0, -269.5)	-535.0(-1738, -7.0)	0.029

Continuous variables were present as mean ± SD or median and inter-quartile range, and the counts were expressed as a percentage.Chi-square test for categorical variables and t test or wilcoxon rank sum test for continuous variables. The following tables show the same expression

∆ : Comparison data of Valuables in final value minus initial value on the two patient groups. A decrease is expressed as a negative number while an increase is expressed as a positive number

## Discussion

In most cases, CHF develops slowly owing to the decompensation of cardiac function. Most patients with CHF go through the compensatory stage of cardiac hypertrophy, accompanied by water and sodium retention and increased inter-tissue fluid accumulation. The course of the disease is long, and the prognosis is poor, owing to which CHF is the terminal manifestation of the vast majority of cardiovascular diseases. Related research reveals that the pathogenesis of CHF involves the abnormal activation of the neuroendocrine system and cytokines, leading to ventricular remodelling and the progressive decompensation of chronic CHF [21]. Chronic cardiac insufficiency after CVS may be related to preoperative cardiac function state and left ventricular dysfunction [22]. Based on this, there may be a similar abnormal activation of the neuroendocrine system and cytokines leading to ventricular remodelling. However, CVS may prevent the natural course of the disease and improve cardiac functional status. However, during patient follow-up after surgery, although few patients did not have organic valvular disease or slight valvular disease, they still had chronic cardiac insufficiency (Table 2). The key to treating CHF is to effectively block the activation of the neuroendocrine system and reduce the occurrence of ventricular remodelling [23, 24].

The present results suggest that sacubitril/valsartan treatment improved cardiac function, ventricular remodelling, LVEF, reduced LVEDD, and LVESD in patients who underwent CVS. The active neprilysin inhibitor sacubitrilat can inhibit the degradation of natriuretic peptide, dilate blood vessels, maintain the balance of water and sodium ions, and improve ventricular remodelling. It is also beneficial in reducing cardiac load and improving myocardial remodelling. Valsartan can inhibit the binding of angiotensin II and angiotensin I receptors, block the physiological activity of angiotensin II, thereby reducing the toxic effect of norepinephrine on cardiomyocytes, and reduce the proliferation and hypertrophy of vascular smooth muscle and cardiomyocytes [25–29]. By competitively inhibiting the biological effects of angiotensin II, valsartan can reduce the cardiac preload, cardiac afterload, and ventricular wall tension. It can also inhibit myocardial cell apoptosis, cardiac hypertrophy, cardiovascular fibrosis, sympathetic hyperactivation, and the release of inflammatory factors and restoreexcitation–contraction coupling [30, 31]. Thus, sacubitril/valsartan can relax blood vessels, reduce platelet aggregation, inhibit fibrin dissolution, control smooth muscle cell migration and proliferation, and reduce sympathetic nerve excitability. By dilating blood vessels and discharging sodium diuresis, the drug combination can effectively improve heart function and prevent the progression of HF [23]. In patients who underwent CVS, there was no organic valvular disease. Patients presented only with CHF, and the pathogenesis may be similar to that of patients with CHF without heart surgery. Therefore, sacubitril/valsartan can be routinely used for patients with CHF after CVS.

The present results showed that sacubitril/valsartan alleviated the symptoms of CHF and significantly reduced NT-proBNP. Sacubitril/valsartan has a significant double-target regulatory effect. On the one hand, it can significantly inhibit the activation of RAAS by the angiotensin II receptor, and on the other hand, it can improve the level of guanine nucleosides through a neprilysin inhibitor, thereby reducing the level of neuroendocrine factors and alleviating the condition of chronic HFrEF. By acting on the RASS and the NPs, sacubitril/valsartan can reduce the secretion of aldosterone, sympathetic nerve activity, cardiac hypertrophy and myocardial fibrosis, and the degradation of NT-proBNP [17, 18, 32].

The present results, together with those of existing reports, demonstrate that sacubitril/valsartan can reduce SBP and DBP. Related randomised controlled trial studies have revealed that sacubitril/valsartan significantly increases urinary sodium excretion and significantly lowers arterial blood pressure compared with angiotensin receptor blockers, with a greater decrease in blood pressure at night than during the day [33, 34]. However, symptomatic hypotension was not observed in this study. The NPs, RAAS, sympathetic nervous system, endothelial function, and immune system regulate blood pressure [35–37]. Sacubitril/valsartan is a dual-acting ARNI in a single molecule. It functions as an angiotensin-receptor blocker via its valsartan molecular moiety and a neprilysin inhibitor via its sacubitril molecular moiety [34]. This suggests that the enhancement of NPs through neprilysin inhibition is an effective approach to improve the BP-lowering effect associated with RAS inhibition in patients with a low-or less-responsive RAS (e.g. salt-sensitive or elderly patients with hypertension) [38]. The NPs have been used as new targets against hypertension, participating in multiple aspects of cardiovascular homeostasis. Natriuretic peptides can discharge natriuretic diuresis and reduce blood volume. They can promote vasodilation and resist vasoconstriction. They also inhibit RAAS and the sympathetic nervous system, which regulates blood pressure [39, 40]. Reducing blood pressure reportedly lowers the risk of new onset of heart failure by as much as 40%, similar to the effect of lowering blood pressure after stroke and greater than the effect of lowering blood pressure during the risk of myocardial infarction [41, 42].

Although sacubitril/valsartan demonstrates beneficial effects against CHF, the following study had certain limitations. The sample size of this study was small, and there was a lack of non-surgical patients with CHF, which is the focus of a prospective, double-blind controlled study. Patients have different sensitivities to drugs, and their basic blood pressure is different. Therefore, the dosage of drugs cannot be standardised but needs to be changed based on the actual conditions of patients, which may result in some bias in our research results.

## Conclusions

In conclusion, based on the reported results, sacubitril/valsartan has potential as a treatment for patients with CHF after CVS. It can improve the cardiac function of such patients, increase their LVEF, and improve ventricular remodelling. Thus, Sacubitril/valsartan has good clinical efficacy and safety in patients with CHF after CVS, which is worthy of active promotion in clinical practice.

## Data Availability

The data of this study will not be shared publically, but Liangwan Chen can be contacted if someone wants the data.

## List of Abbreviations

ACEI: Angiotensin-converting enzyme inhibitor
ARB: Angiotensin receptor blocker
ARNI: Angiotensin receptor - neprilysin inhibitor
BP: Blood pressure
CHF: Chronic heart failure
CVS: Cardiac valve surgery
DBP: Diastolic blood pressure
HF: Heart failure
HFrEF: Heart failure with reduced ejection fraction
LVEDD: Left ventricular end-diastolic diameter
LVEF: Left ventricular ejection fraction
LVESD: Left ventricular end-systolic diameter
NPS: Natriuretic peptide system
NYHA: New York Heart Association
NT-proBNP: N-terminal prohormone of B-type natriuretic peptide
RAAS: Renin–angiotensin–aldosterone system
RAS: Renin angiotensin system
SBP: Systolic blood pressure
